# Differentiating COVID-19 and influenza in children: hemogram parameters as diagnostic tools

**DOI:** 10.3389/fpubh.2024.1377785

**Published:** 2024-07-11

**Authors:** Ramazan Dulkadir, Bahar Oztelcan Gunduz

**Affiliations:** ^1^Ahi Evran University, Kırşehir, Türkiye; ^2^Gulhane Training and Research Hospital, Ankara, Türkiye

**Keywords:** COVID-19, influenza, laboratory parameters, differences, COVID-19 pandemic

## Abstract

**Introduction:**

It is not always possible to differentiate between influenza and COVID-19 based on symptoms alone. This is a topic of significant importance as it aims to determine whether there are specific hematological parameters that can be used to distinguish between influenza and COVID-19 in children.

**Methodology:**

Two hundred thirty-one children between the ages of 1 month and 18 years who presented to the children’s outpatient clinic between June 2021 and June 2022 with similar symptoms and were tested with an influenza test and a COVID-19 PCR test were included in the study. Of the patients included in the study, 130 tested positive for COVID-19 and 101 positive for influenza. The patients were evaluated for hematological parameters.

**Results:**

Age, eosinophils and monocyte factors were shown to be statistically significantly effective in COVID-19. The risk of COVID-19 increased 1,484-fold with age, 10,708-fold with increasing eosinophil count, and 1,591-fold with increasing monocyte count. The performance of the monocyte count and eosinophil count was assessed by receiver operating characteristic curve (ROC) analysis. According to the performed ROC analysis, the area under the curve (AUC) value was observed to be 0.990 for monocytes. According to the cutoff point >1.50, the sensitivity value was determined as 98.4% and the specificity value as 97.0%. AUC significance for eosinophils was found to be 0.989. According to the cutoff point >0.02, the sensitivity value was determined as 99.2% and the specificity value as 93.1%.

**Conclusion:**

In the diagnosis of COVID-19, the eosinophil count and monocyte count are easily accessible, inexpensive, and important parameters in terms of differential diagnosis and can help in the differentiation of COVID-19 from influenza during seasonal outbreaks of the latter. Developing parameters for clinicians to use in diagnosing COVID-19 and influenza can facilitate their work in practice.

## Introduction

Influenza (flu) and COVID-19 are droplet-borne pathogens that affect the respiratory system. Both viruses are enveloped RNA viruses. In addition, there are divisions between them in the terminology of the incubation period, the risk groups they affect, and the speed of infection. It may not always be likely to distinguish between influenza and COVID-19 just by examining the symptoms. With COVID-19 and influenza, the period from when the individual is infected until the onset of symptoms is one or more days. If the patient is infected with COVID-19, the symptoms may take longer to grow compared to influenza. While influenza symptoms last 1–4 days after infection, for COVID-19 this period can be 2–14 days. Also, in both COVID-19 and influenza, the virus can be transmitted before symptoms appear.

Influenza in children is well documented and associated with serious complications, including death (1, 2). However, pediatric data on COVID-19 are still at a limited level. Compared to COVID-19 infected adults, studies have found that hospitalizations in children are less common, symptoms such as fever, cough and shortness of breath are less perceived (3, 4) and pediatric deaths relevant to COVID-19 are rare (5). Nevertheless, publications on a clinical picture in children called multisystem inflammatory syndrome, which is rare but can be fatal, keep popping up (6).

Laboratory findings in children with COVID-19 demonstrate similarities to other viral respiratory infections (7). It is typically observed that the leukocyte count remains within the normal range, with a decrease in either neutrophil or lymphocyte count. Additionally, thrombocytopenia may occur. However, it is important to note that in the comparison between COVID-19 cases and influenza B cases, there was a significantly higher occurrence of leukocytosis in the COVID-19 cases (8).

Testing is needed to identify the disease and ensure diagnosis in these two infections with similar symptoms. Rapid determination of the etiology of both infections is particularly important when immediate action is required. Taking isolation and quarantine measures for COVID-19 and starting influenza treatment promptly are important to halt the progression of the infections.

To date, very few studies have been published directly comparing pediatric cohorts of COVID-19 patients with seasonal influenza patients. Distinguishing between influenza and COVID-19, guiding patient care, and supporting a comprehensive COVID-19 control program are important to isolating cases, quickly identifying those who have been contacted, and taking action, including quarantine. Assuming that preventing COVID-19 would be more difficult at a time when influenza and other respiratory diseases are beginning to increase in society, especially as seasonal influenza approaches, the hematological discrepancies in the instruction were considered study to distinguish these infections from others could be of practical importance. Many newer approaches are being developed today that are appreciated to provide analysis and control of blood hematological parameters as simple, effective, and rapid laboratory procedures that can be recalled by physicians charged with evaluating infectious inflammatory responses. The study aimed to determine if there is a parameter to distinguish between influenza and COVID-19 by using specific hematological parameters.

## Clinical methodology

A total of 231 children between the ages of 1 month and 18 years who presented to the children’s outpatient clinic at Kirsehir Training and Research Hospital between June 2021 and June 2022 with comparable symptoms were included in the study. The cases with known chronic infections and hematological diseases were excluded from the study. The sample selection process involved identifying children who visited the children’s outpatient clinic during the specified time frame and had symptoms such as fever, cough, nasal discharge, fatigue, and malaise. Children meeting these criteria were considered eligible for inclusion in the study.

Blood count, influenza and COVID-19 test data were retrieved and recorded by the hospital’s automation system. The cases were tested in line with the recommendations of the producers through Humasis Influenza Antigen Card Plus (Korea), SD Biosensor Standard-Q Influenza A/B (Korea) and SD Biosensor Standard-F Influenza A/B FIA (Korea) rapid antigen detection kits (sensitivity 87%/93% specificity 95%/98%). The diagnoses of the patients suspected with COVID-19 who applied to our hospital were confirmed with SARS CoV-2 PCR test. Viral RNAs extracted from nasopharyngeal swab samples through vNAT solution were studied with Bio-speedy SARS-CoV-2 RT-qPCR kits, which are one-step reverse transcription (RT) and real-time PCR (qPCR) (RT-qPCR) kits that target ORF1ab and N gene fragments in Bio-Rad CFX96 Touch TM device [When used with vNAT isolation method, the limit of determination (LoD) of the kit is 200 genome/mL for nasopharyngeal aspirate/lavage and phlegm samples, 281 genome/mL for bronchoalveolar lavage samples, 562 genome/mL for oropharyngeal swabs, 200 genome/mL for nasopharyngeal Dacron swabs, and 89 genome/mL for nasopharyngeal polyester swabs]. The samples taken from the patients included in the study were appropriately placed in tubes that contained Bio-Speedy viral nucleic acid tampon (vNAT), and the tubes were delivered to the laboratory in the shortest time possible. Viral RNAs extracted with vNAT solution were studied with Bio-speedy SARS-CoV-2 RT-qPCR kits in Bio-Rad CFX96 Touch TM device (In line with the kit recommendations, the threshold value for the Bio-Rad CFX 96 device was set at 200 RFU). RT-qPCR, which targeted ORF1ab and N gene fragments, was performed. The samples were studied with oligonucleotide kit (internal control), which targets human RNase P gene, in order to check nucleic acid extraction and inhibition. Internal control (IC) (RNase P) curve was marked blue, while SARS-CoV-2 (N) and SARS-CoV-2 (ORF1ab) curve were marked red. The internal control curve being a sigmoidal curve shows that there was no problem in the sampling and isolation steps. When SARS-CoV-2 curve was sigmoidal, the sample was reported as POSITIVE, and when it was not sigmoidal, the sample was reported as NEGATIVE.

### Ethical approval

The ethical board approval for the study was obtained from Ahi Evran University Faculty of Medicine Ethics Committee with decision number 2022–16/120. The study was conducted in line with the ethical principles set by Helsinki Declaration.

### Statıstical methodology

The data were analyzed through IBM SPSS Statistics Standard Concurrent User V 26 (IBM Corp., Armonk, New York, ABD) and MedCalc^®^ Statistical Software version 19.6 (MedCalc Software Ltd., Ostend, Belgium) statistical software. Descriptive statistics were presented as number of unit (*n*), percentage (*%*), median (*M*), and interquartile range (*IQR*) values. The normal distribution of numerical variables was evaluated with Shapiro–Wilk normality test. In the comparison of groups by numerical variables, Mann–Whitney *U* test was employed. The groups were compared in terms of gender through Chi-square test. Factors effective on COVID-19 were analyzed through simple and multiple binary logistic regression by correcting according to age. The factors in the final model were determined through backward Wald elimination method. The performances of monocyte and eosinophil in predicting COVID-19 disease was evaluated with ROC analysis. *p* < 0.05 was accepted as statistically significant.

## Results

In the study, a total of 231 patients, 130 COVID-19 and 101 influenza patients, were included in the study. The number of boys in the COVID-19 group was 71 (54.6%) and 57 in the influenza group (56.4%). No statistically significant difference between the groups was found with regard to gender distribution (*p* > 0.791). The mean age of the COVID-19 patients was 17 years old, while it was 5 years old in the influenza group, showing a statistically significant difference (*p* < 0.001). The difference in the leukocyte counts of the groups was not statistically significant. The RBC, HGB, HCT, MCV, MCH, and MCHC levels of the COVID-19 patients were statistically higher than those of the influenza patients. It was found that the RDW and PLT values of the influenza patients were statistically significantly higher than those of the patients in the COVID-19 group. The neutrophil, lymphocyte and monocyte counts of the COVID-19 patients were statistically higher than those of the influenza patients. The difference between the groups in lymphocyte/neutrophil and lymphocyte/monocyte rates was not statistically significant. It was found that the eosinophils, basophils, MPV and PDW levels were statistically higher in the COVID-19 patients. The difference between the groups in terms of PCT, NRBC and NRBC-I values was not statistically significant. Finally, IG and IG-I scores were determined to be statistically significantly higher in the influenza group (Table 1).

**Table 1:** Comparison of the variables in the study by groups.

	Groups		Test statistics	
	COVID-19 *n* = 130	Influenza *n* = 101	Test value	*p-*value
Gender *n* (%)				
Female	59 (45.4)	44 (43.6)	0.076^‡^	0.791
Male	71 (54.6)	57 (56.4)		
Age	17.00 (3.00)	5.00 (4.00)	11.629^†^	**<0.001**
CRP (0.1–2.8 mg/L)	3.00 (7.00)	4.00 (9.75)	2.379^†^	**0.017**
WBC (6.0–17.0 10^3^/uL)	6.35 (2.72)	6.90 (4.08)	1.651^†^	0.099
RBC (3.6–5.8 10^6^/uL)	5.19 (0.75)	4.83 (0.54)	4.668^†^	**<0.001**
HGB (10.7–13.0 g/dL)	14.35 (2.63)	12.70 (1.30)	7.625^†^	**<0.001**
HCT (33–45%)	42.80 (7.15)	38.55 (3.90)	6.903^†^	**<0.001**
MCV (81.8–95.5 FL)	82.65 (5.58)	80.50 (6.47)	3.940^†^	**<0.001**
MCH (27–34 pg)	28.00 (2.60)	26.60 (2.60)	5.915^†^	**<0.001**
MCHC (32.0–36.0 g/dL)	33.70 (1.40)	32.80 (1.80)	5.310^†^	**<0.001**
RDW (11.5–15%)	12.40 (1.05)	13.10 (1.70)	5.451^†^	**<0.001**
PLT (130–450 10^3^/uL)	244.0 (86.7)	260.0 (85.0)	2.046^†^	**0.041**
NEU (41–72%)	54.50 (17.33)	3.61(3.68)	12.474^†^	**<0.001**
LYM (18.5–46%)	32.90 (21.45)	2.21 (1.77)	12.266^†^	**<0.001**
MONO (4.3–12.0%)	9.90 (5.28)	0.73 (0.47)	12.571^†^	**<0.001**
L/N	0.63 (0.63)	0.57 (1.00)	0.052^†^	0.959
L/M	3.42 (3.05)	2.73 (2.99)	1.048^†^	0.295
EOS (0.0–5.0%)	1.10 (1.60)	0.03 (0.08)	12.123^†^	**<0.001**
BASO (0–2%)	0.30 (0.30)	0.02 (0.03)	12.526^†^	**<0.001**
MPV (7.8–12 FL)	10.40 (1.20)	9.45 (1.00)	6.991^†^	**<0.001**
PCT (0–0.99%)	0.25 (0.08)	0.25 (0.09)	0.523^†^	0.601
PDW (0–99 FL)	11.70 (2.55)	9.90 (2.00)	7.341^†^	**<0.001**
NRBC (0%)	0.00 (0.00)	0.00 (0.00)	1.076^†^	0.282
IG (0.0–0.8%)	0.20 (0.10)	0.30 (0.20)	4.064^†^	**<0.001**

Data are given as median (interquartile range) or *n* (%), ^‡^Chi-square test, ^†^Mann–Whitney *U* test, Comparisons with values in bold are statistically significant. WBC, white blood cells; MPV, mean platelet volume; PDW, platelet distribution width; RBC, red blood cells; MCH, mean corpuscular hemoglobin; MCHC, mean corpuscular hemoglobin concentration; Hb, hemoglobin; Plt, Platelet; MCV, mean corpuscular volume; NEU, neutrophil; LYM, lymphocyte; MONO, monocyte; BASO, basofil; EOS, eosinophils; CRP, C-reactive protein; L/M, lymphocyte-to-monocyte ratio; L/N, lymphocyte-to-neutrophil ratio.

Since the comparisons in Table 1 showed a statistically significant difference between the groups with respect to age, taking into account that other variables in the study would be affected by the age factor, age-corrected factors were evaluated by a simple logistic regression analysis to determine them identify the factors effective on COVID-19 and presented in Table 2. According to the results of simple logistic regression analysis presented in Table 2, MCHC, neutrophils, lymphocytes, monocytes, lymphocytes/monocytes, eosinophils, basophils, MPW, PDW, IG, and IG-I factors were each found to be statistically significantly effective at COVID-19 observed.

**Table 2:** Simple logistic regression analysis of the effect of age-corrected variables on COVID-19.

	*p*	OR	95% C.I. for OR
CRP (0.1–2.8 mg/L)	0.179	1.016	0.993–1.039
WBC (6.0–17.0 10^3^/uL)	0.793	0.980	0.841–1.141
RBC(3.6–5.8 10^6^/uL)	0.649	0.775	0.258–2.324
HGB (10.7–13.0 g/dL)	0.856	0.968	0.678–1.382
HCT (33–45%)	0.447	0.961	0.866–1.065
MCV (81.8–95.5 FL)	0.110	0.927	0.844–1.017
MCH (27–34 pg)	1.000	1.000	0.815–1.227
MCHC (32.0–36.0 g/dL)	**0.019**	1.492	1.068–2.083
RDW (11.5–15%)	0.655	0.917	0.628–1.340
PLT (130–450 10^3^/uL)	0.657	1.001	0.995–1.007
NEU (41–72%)	**<0.001**	1.155	1.102–1.210
LYM (18.5–46%)	**<0.001**	1.172	1.110–1.238
MONO (4.3–12.0%)	**<0.001**	2.103	1.637–2.700
L/N	0.165	1.125	0.953–1.329
L/M	**<0.001**	1.425	1.180–1.721
EOS (0.0–5.0%)	**<0.001**	125.470	18.279–861.241
BASO (0–2%)	**<0.001**	1.227	1.145–1.315
MPV (7.8–12 FL)	**0.036**	1.763	1.038–2.996
PCT (0–0.99%)	0.057	1.068	0.998–1.143
PDW (0–99 FL)	**0.048**	1.321	1.002–1.741
NRBC (0%)	0.928	1.269	0.007–227.638
IG (0.0–0.8%)	**<0.001**	0.948	0.921–0.976

β, regression coefficient; s.e., standard error of β; OR, odds ratio; C.I., confidence Interval. WBC, white blood cells; MPV, mean platelet volume; PDW, platelet distribution width; RBC, red blood cells; MCH, mean corpuscular hemoglobin; MCHC, mean corpuscular hemoglobin concentration; Hb, hemoglobin; Plt, platelet; MCV, mean corpuscular volume; NEU, neutrophil; LYM, lymphocyte; MONO, monocyte; BASO, basofil; EOS, eosinophils; CRP, C-reactive protein; L/M, lymphocyte-to-monocyte ratio; L/N, lymphocyte-to-neutrophil ratio. Bold values indicate significant.

To determine the final factors effective on COVID-19, variables with a value of *p* < 0.20 in Table 2 were included in the multiple logistic regression analysis. To determine the final model, the Backward-Wald method was used as the elimination method. The age variable was kept constant at each step until the final model. The final model included factors with a value of *p* < 0.05. According to Table 3, age, eosinophils and monocyte factors were statistically significantly effective in COVID-19.

**Table 3:** Multiple logistic regression analysis of the variables found to be effective of COVID-19.

	*β*	s.e.	Wald	*p*	OR	95% C.I. for OR
Constant	−7.689	1.582	23.608	<0.001	0.001	
Age	0.394	0.111	12.630	<0.001	1.484	1,194–1.844
Eosinophil	2.371	0.985	5.797	0.016	10.708	1.554–73.785
Monocyte	0.464	0.134	12.021	0.001	1.591	1.224–2.069

β, regression coefficient; s.e, standard error of β; OR, odds ratio; C.I., confidence interval. Variables entered on step 1: CRP, MCV, MCHC, NEU, L/N, LYM, L/M, MONO, EOS, BASO, MPV, PCT, PDW, IG. Elimination method: Backward Wald. Hosmer–Lemeshow goodness-of-fit: Chi-square = 6.808; *p* = 0.557. Cox and Snell R Square = 0.703.

According to the results in the Table 3, the risk of COVID-19 increased 1.484-fold with age, 10.708-fold with increasing eosinophil count, and 1.591-fold with increasing monocyte count. According to Homer-Lemeshow statistics, the established model achieved a goodness of fit. The variables in the model explained the dependent variable 70.3%.

In Table 4, the performances of monocytes and eosinophils in predicting COVID-19 disease were evaluated using Receiver Operating Characteristic Curve (ROC) analysis.

**Table 4:** ROC curve analysis results for age-corrected lymphocyte and lymphocyte/monocyte.

	AUC (95% CI)	*p*	Cutoff point	Sensitivity (95% CI)	Specificity (95% CI)
Monocyte	0.990 (0.968–0.999)	<0.001	>1.50	98.4 (94.6–99.8)	97.0 (91.6–99.4)
Eosinophil	0.989 (0.966–0.998)	<0.001	>0.02	99.2 (95.8–100.0)	93.1 (86.2–97.2)

AUC, area under the curve; CI, confidence interval.

**Figure 1:**
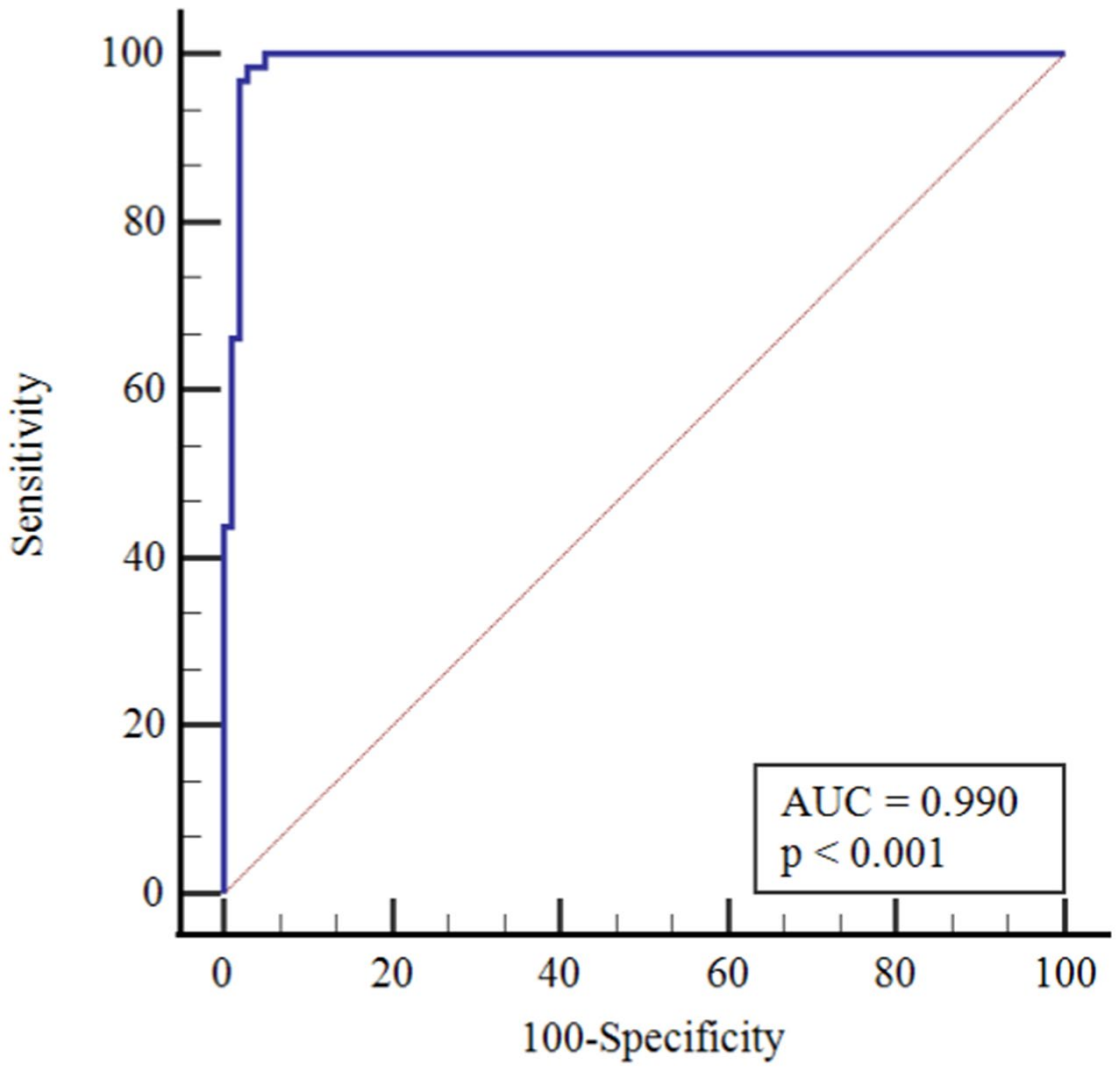
ROC Curve for age-corrected monocyte.

**Figure 2:**
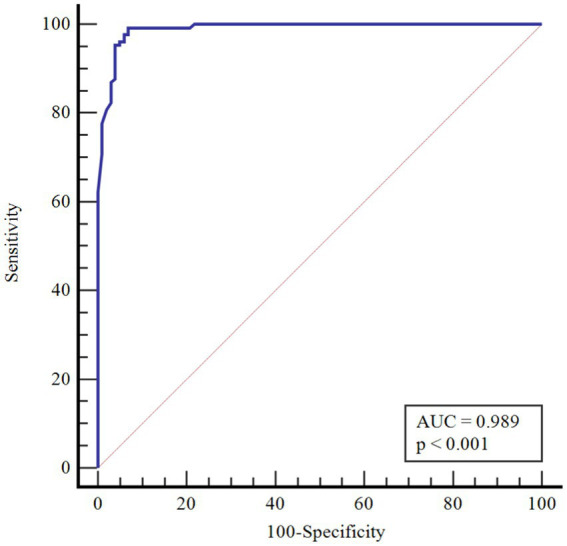
ROC Curve for age-corrected eosinophil.

**Figure 3:**
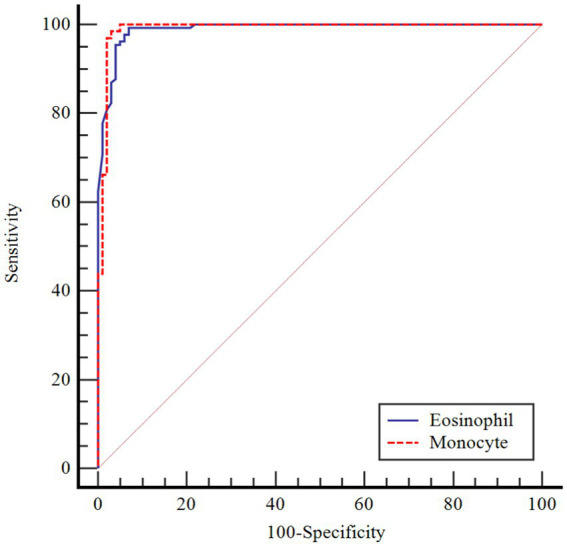
ROC Curve for age-corrected monocyte and eosinophil.

According to ROC analysis, the area under the curve (AUC) value is 0.990 for monocytes. According to the optimal cutoff point >1.50, the sensitivity value was determined as 98.4% and the specificity value as 97.0%. The AUC value for eosinophils is 0.989. According to the optimal cutoff point >0.02, the sensitivity value was determined as 99.2% and the specificity value as 93.1% (Figures 1–3).

## Discussion

Influenza and COVID-19 are two different respiratory pathogens that can be found in children, cause hospitalizations, and share similarities and differences.

The first difference we defined in this study was the variation in age groups. Patients diagnosed with COVID-19 were older than those diagnosed with influenza. In a survey conducted in China that examined 72,314 pediatric cases, COVID-19 was found in only 1% of children under the age of 10 (7), and in another survey that examined 2,135 pediatric cases, the median age for COVID-19 was 19 will definitely be 7 years (9). In a study of 11,535 pediatric cases conducted in Turkey, the median age of COVID-19 was found to be 8 years. In the present study, the median age for COVID-19 was determined as 17 (IQR 3), while the mean age of influenza-positive pediatric cases was 5 years. In 2019–2020 influenza season, the mean age for influenza was found as 3.91 ± 3.3 (10), and in yet another study, the median age was found as 6.2 years (11). The difference found in the age group distributions of children in regard to COVID-19 and influenza in the present study and in the literature can help us make a simple distinction between influenza and COVID-19 in this period when the influenza season is approaching.

Data on children with COVID-19 show that it does not result in abnormal red blood cell (RBC) counts and hemoglobin (Hb) levels (12–15) and that anemia is prevalent in children with Kawasaki or related diseases (16). This is in contrast to previous studies and can be interpreted for a variety of reasons. When compared to influenza cases in our study, it was observed that RBC, HGB, HCT, MCV, MCH, and MCHC levels of COVID-19 patients were statistically significantly higher than those of the influenza cases. Starting in the respiratory tract, COVID-19 disease can affect numerous systems, including the hematopoietic system. It is known that changes in hematological parameters in particular affect the severity and course of the disease. There is a wealth of evidence showing that platelet counts decrease, notoriously in severe cases (17).

The evaluation included a comparison between the results of this investigation and published work. It was found that many hematological parameters, especially PLT, WBC, RBC, Hb, and RDW, have changed significantly and that influenza infection has triggered an increased neutrophil count and decreased lymphocyte count (18, 19). The reason that the hemoglobin and MCV values of the COVID-19 patients included in the present study are statistically different from those of the influenza patients could be due to the higher median age of the COVID-19 patients.

While the laboratory abnormalities most commonly reported in the literature are lymphopenia, eosinopenia, monocytosis, and high CRP levels (20), eosinophil and monocyte counts were quite high in COVID-19 patients compared to influenza patients in our study, particularly those Threat of COVID-19 increased 10,708-fold when eosinophil count increased and 1,591-fold when monocyte count increased. We may not be able to illustrate how a rationale for the high frequency of eosinophils in our COVID-19 patients was found, suggesting that SARS-CoV-2 is directly or indirectly responsible for eosinophils as a result of infection or recovery is responsible. Increases in neutrophil, lymphocyte, and neutrophil/lymphocyte rates have been reported in studies conducted on children with COVID-19 and healthy children (21).

A notable feature of this research was the observation that eosinophil and monocyte determinants were statistically significantly effective in COVID-19. We found that the threat of COVID-19 increased 1,484-fold with age, 10,708-fold with increasing eosinophil count, and 1,591-fold with increasing monocyte count. In a study conducted on adults, it was observed that eosinophil counts and rates were significantly lower in COVID-19 patients with critical illness than in patients with moderate and severe illness (22). In the identical study, it was shown that eosinophil counts and rates were significantly and inversely correlated with markers of infection; That is, as C-reactive protein (CRP), procalcitonin (PRO), and ferritin (FER) increased, eosinophil levels decreased and the progressive reduction in eosinophil counts and rates worsened the viral infection (22). Eosinopenia was noted in hospitalized COVID-19 patients in early publications on COVID-19 relevant studies and this scenario has been associated with the intensity of infection or a poor prognosis (23–28). According to the literature, although abnormal and large macrophage inflammation was found in lung biopsies and BAL (bronchoalveolar lavage) samples from COVID-19 patients, eosinophils were absent, and thus it is complicated to say that eosinophils are a specific finding of COVID-19 infection (29–31). However, the fact that eosinophil levels were restored in patients prior to discharge suggests that this scenario may be indicative of restoration of clinical criteria of eosinophil resolution (32). Studies conducted have shown that eosinophils in the airways were subject to rapid viral clearance in allergy and influenza infection, that eosinophils in particular played a role in anti-influenza immunity, and that they provided protection against airway damage (32, 33). In addition, an increase in serum IL-5 levels and peripheral eosinophilia was found in the pediatric population with acute pneumonia relevant to influenza viruses (34). It is not known if eosinophilia is an indicator of an excessive immune response or if it is a rare immune response that occurs during an infection-induced cytokine storm. A very high frequency of eosinophilia is uncommon in non-COVID-19 patients, and such a high incidence has never been documented in other viral infections such as influenza (35).

One of the very important variations in the difference between COVID-19 and influenza defined in our study was the significantly increased monocyte count in COVID-19 cases compared to children with influenza infection.

In a study by Tiwari et al. (36) the monocyte count was normal in 81.8% of pediatric cases, while mild monocytosis was observed in only 18.2% of cases.

There is partial information showing monocytosis in pediatric cases. On the other hand, in a study by Henry et al. (37) 3,377 adult patients were shown to have reduced monocyte counts with COVID-19 infection, and Xiong et al. (38) reported monocytosis in children with asymptomatic COVID-19 compared to children with symptomatic COVID-19. In a cohort study, in peripheral blood smear of children with COVID-19, 36.7% of neutrophils showed hypergranulation/lobulation abnormality and 13.3% of monocytes showed vacuole defects compared to COVID-19 negative cases (39). Laboratory markers for COVID-19 are not specific and their use in clinical practice is limited. Many studies have been published that identify poor prognosis with relevant lymphopenia and hypercoagulability in adults with severe disease (40). However, pediatric studies are still at a limited level. The performance of monocytes and eosinophils in predicting COVID-19 infection was assessed in this study for the first time by Receiver Operating Characteristics Curve (ROC) analysis. According to the ROC analysis performed, the area under the curve (AUC) value for monocytes was 0.990. According to the cutoff point >1.50, the sensitivity value was 98.4% and the specificity value was 97.0%. The AUC value for eosinophils was 0.989. According to the cutoff point >0.02, the sensitivity value was determined as 99.2% and the specificity value as 93.1%.

Limitations of the study, the study had a limited number of participants, with a sample size of 231. Studies with larger sample sizes would provide more reliable results. The study was conducted at a single children’s outpatient clinic. Multi-center studies including children from different regions and healthcare facilities would allow for better generalizability. By addressing these limitations and implementing the suggested recommendations, future studies can contribute to a better understanding of differentiating between COVID-19 and influenza in children and improve diagnostic capabilities in clinical practice.

## Conclusion

It is believed that this distinction can be considered an important laboratory marker when the upcoming influenza season and the potential for a recent peak in COVID-19 are taken into account. Additionally, it is expected that as the number of children tested for COVID-19 increases, the difference between the determinants of both infections will become clearer. Future research in this specific area can focus on addressing these limitations and exploring additional aspects; Comparison of the performance and sensitivity of different COVID-19 test methods, such as PCR, rapid antigen tests, and serological tests, in pediatric populations. Longitudinal studies to track the progression and outcomes of influenza and COVID-19 in children over time, considering factors such as age, underlying medical conditions, and vaccination status. Exploration of potential biomarkers or additional laboratory markers that can aid in distinguishing between influenza and COVID-19 infections in children.

## Data availability statement

The original contributions presented in the study are included in the article/supplementary material, further inquiries can be directed to the corresponding author.

## Ethics statement

The ethical board approval for the study was obtained from Ahi Evran University Faculty of Medicine Ethics Committee with decision number 2022–16/120. The study was conducted in line with the ethical principles set by Helsinki Declaration. The studies were conducted in accordance with the local legislation and institutional requirements. Written informed consent for participation in this study was provided by the participants’ legal guardians/next of kin. Written informed consent was obtained from the individual(s), and minor(s)’ legal guardian/next of kin, for the publication of any potentially identifiable images or data included in this article.

## Author contributions

RD: Conceptualization, Data curation, Formal analysis, Funding acquisition, Methodology, Writing – original draft, Writing – review & editing. BO: Conceptualization, Resources, Validation, Writing – review & editing.
